# Mitochondrial Dysfunction Induced by *N*-Butyl-1-(4-Dimethylamino)Phenyl-1,2,3,4-Tetrahydro-β-Carboline-3-Carboxamide Is Required for Cell Death of *Trypanosoma cruzi*

**DOI:** 10.1371/journal.pone.0130652

**Published:** 2015-06-18

**Authors:** Hélito Volpato, Vânia Cristina Desoti, Rodrigo Hinojosa Valdez, Tânia Ueda-Nakamura, Sueli de Oliveira Silva, Maria Helena Sarragiotto, Celso Vataru Nakamura

**Affiliations:** 1 Programa de Pós-Graduação em Ciências Biológicas—Biologia Celular e Molecular, Universidade Estadual de Maringá, Maringá, Paraná, Brazil; 2 Programa de Pós-Graduação em Ciências Farmacêuticas, Universidade Estadual de Maringá, Paraná, Brazil; 3 Departamento de Farmácia, Instituto Federal do Paraná, Palmas, Paraná, Brazil; 4 Departamento de Química, Universidade Estadual de Maringá, Maringá, Paraná, Brazil; Albert Einstein College of Medicine, UNITED STATES

## Abstract

**Background:**

Chagas’ disease is caused by the protozoan *Trypanosoma cruzi *and affects thousands of people worldwide. The available treatments are unsatisfactory, and new drugs must be developed. Our group recently reported the trypanocidal activity of the synthetic compound N-butyl-1-(4-dimethylamino)phenyl-1,2,3,4-tetrahydro-β-carboline-3-carboxamide (**C4**), but the mechanism of action of this compound was unclear.

**Methodology/Principal Findings:**

We investigated the mechanism of action of **C4** against epimastigote and trypomastigote forms of *T*. *cruzi*. The results showed alterations in mitochondrial membrane potential, alterations in cell membrane integrity, an increase in the formation of reactive oxygen species, phosphatidylserine exposure, a reduction of cell volume, DNA fragmentation, and the formation of lipid inclusions.

**Conclusion/Significance:**

These finding suggest that mitochondria are a target of **C4**, the dysfunction of which can lead to different pathways of cell death.

## Introduction

Chagas’ disease is a tropical infection caused by *Trypanosoma cruzi*. Approximately 7–8 million people worldwide are infected by this protozoan, mostly in Latin America. Up to 30% of chronically infected individuals develop cardiac complications [1]. It is found endemically in 21 Latin American countries, and 28 million people are at risk of acquiring this infection around the world [2].

**Fig 1:**
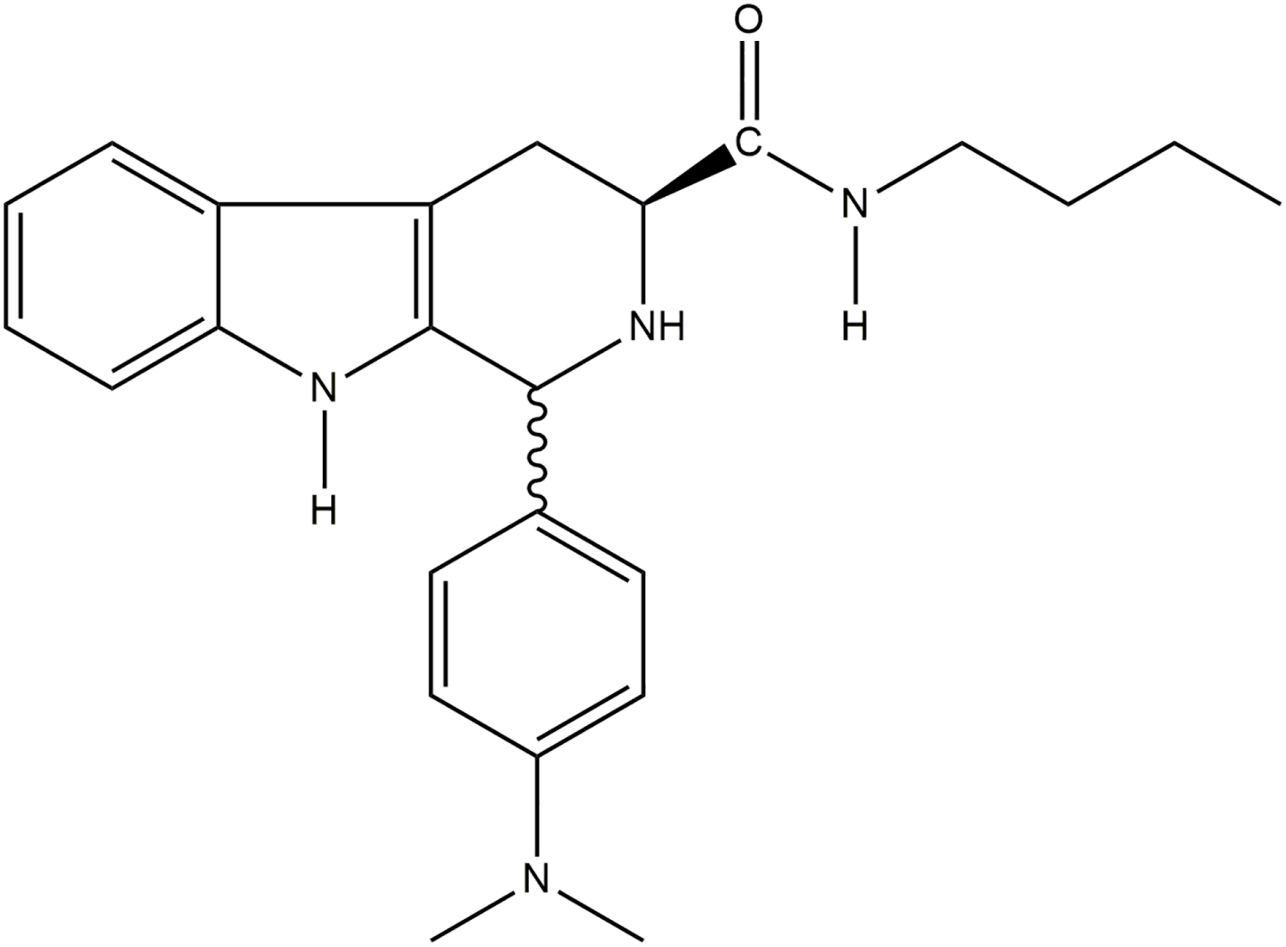
Chemical structure of *N*-butyl-1-(4-dimethylamino)phenyl-1,2,3,4-tetrahydro-β-carboline-3-carboxamide (C4).

The available treatment for Chagas’ disease is based on only two drugs, nifurtimox and benznidazole, which were discovered approximately 40 years ago. Both drugs are only partially effective and have many side effects [3, 4]. The search for new drugs must be intensified. Different research groups are investigating the effectiveness of possible trypanocidal agents [5]. Our group demonstrated the *in vitro* and *in vivo* effects on *T*. *cruzi* of some β-carboline compounds, especially *N*-butyl-1-(4-dimethylamino)phenyl-1,2,3,4-tetrahydro-β-carboline-3-carboxamide (**C4)** (Fig 1) [6, 7]. This compound was effective against the three evolutive forms of *T*. *cruzi*. Furthermore, transmission electron microscopy indicated that the mitochondrion is the major organelle affected by this compound in trypanosomatids, such as *T*. *cruzi* and *Leishmania amazonensis* [6, 8]. This compound has also been shown to have low toxicity in mammalian cells *in vitro* and other animal models [6, 7].

The present study evaluated biochemical alterations in epimastigote and trypomastigote forms of *T*. *cruzi* treated with **C4**. Flow cytometry, fluorimetry, and fluorescence microscopy were used to investigate cellular and subcellular structures and identify organelles that are affected by **C4** treatment. We found that mitochondrial damage may be a possible target for **C4** in these parasites, thus providing a better understanding of the mechanism of action of this compound. Based on our results, we suggest that mitochondrial dysfunction induced by **C4** can lead to different pathways of cell death in *T*. *cruzi*.

## Materials and Methods

### 2.1. Chemicals

Actinomycin D, antimicyn A (AA), carbonyl cyanide *m*-chlorophenylhydrazone (CCCP), 2’,7’-dichlorodihydrofluorescein diacetate (H_2_DCFDA), digitonin, dimethylsulfoxide (DMSO), rhodamine 123 (Rh123), and Nile red were purchased from Sigma-Aldrich (St. Louis, MO, USA). Dulbecco’s modified Eagle’s medium (DMEM) and fetal bovine serum (FBS) were obtained from Invitrogen (Grand Island, NY, USA). Annexin-V FITC, the MitoSOX kit, propidium iodide (PI), and the TUNEL kit were obtained from Invitrogen (Eugene, OR, USA). All of the other reagents were of analytical grade.

### 2.2. Synthesis of C4

**C4** was synthesized as previously described [6].

### 2.3. Substance preparation

**C4** was prepared in DMSO. All of the groups, including the controls were tested at final concentrations of less than 1% DMSO, a concentration that was found not to affect the parasite.

### 2.4. Parasites

The experiments were performed with the Y strain of *T*. *cruzi*. Epimastigote forms were grown in Tryptose Liver Infusion (LIT) supplemented with 10% FBS at 28°C for 96 h. Trypomastigote forms were obtained from the supernatant of an infected LLCMK_2_ cells monolayer (epithelial cell of monkey kidney; *Macaca mulatta*) in DMEM supplemented with 2 mM L-glutamine, 10% FBS, 50 units/mL penicillin, and 0.05 mg/mL streptomycin and buffered with sodium bicarbonate in a 5% CO_2_ air mixture at 37°C. Sub-confluent cultures of LLCMK_2_ cells were infected with 1 × 10^6^ trypomastigotes/mL. Extracellular parasites were removed after 24 h. The cells were washed, and these cultures were maintained in DMEM that contained 10% FBS until trypomastigotes emerged from the infected cells.

### 2.5. Mitochondrial membrane potential

Epimastigotes (5 × 10^6^ cells/mL treated with 18.0 and 77.0 μM of **C4**) and trypomastigotes (1 × 10^7^ cells/mL treated with 45.0 and 230.0 μM of **C4**) of *T*. *cruzi* were incubated at 28°C and 37°C, respectively, for 3 h. Afterward, the parasites were washed and incubated with 5 μg/mL Rh123 for 15 min to verify mitochondrial membrane potential (ΔΨm). CCCP (100.0 μM) was used as a positive control. The data acquisition and analysis were performed using a FACSCalibur flow cytometer (Becton-Dickinson, Rutherford, NJ, USA) equipped with CellQuest software (Joseph Trotter, The Scripps Research Institute, La Jolla, CA, USA). A total of 10,000 events were acquired in the region that was previously established as the one that corresponded to the parasites.

### 2.6. Fluorimetric detection of mitochondrial-derived O_2_^•−^

Epimastigote and trypomastigote forms of *T*. *cruzi* (2 × 10^7^ cells/mL) were harvested and washed with Krebs-Henseleit (KH) solution buffer that contained 15 mM NaHCO_3_, 5 mM KCl, 120 mM NaCl, 0.7 mM Na_2_HPO_4_, and 1.5 mM NaH_2_PO_4_ (pH 7.3). The cells were loaded with 5 μM MitoSOX reagent and incubated for 10 min at room temperature while protected from light. After incubation with MitoSOX reagent, the parasites were washed twice with KH buffer and untreated or treated with 18.0 and 77.0 μM of **C4** (for epimastigotes) and 45.0 and 230.0 μM of **C4** (for trypomastigotes). Antimycin A (10 μM), which is known to induce superoxide anion (O_2_^•−^) production by mitochondria, was used as a positive control. MitoSOX detection was performed using black 96-well plates for 3 h. Fluorescence was measured in a fluorescence microplate reader (Victor X3, PerkinElmer) at an excitation wavelength of 510 nm and emission wavelength of 580 nm [9].

### 2.7. Fluorimetric detection of reactive oxygen species

Epimastigotes (1 × 10^6^ cells/mL treated with 18.0 and 77.0 μM of **C4**) and trypomastigotes (1 × 10^7^ cells/mL treated with 45.0 and 230.0 μM of **C4**) of *T*. *cruzi* were incubated at 28°C and 37°C, respectively, for 24 h. Afterward, the parasites were washed and resuspended in PBS (pH 7.4). Hydrogen peroxide (20 μM) was used as a positive control. Afterward, these parasites were loaded with 10 μM of the cell-permeable probe H_2_DCFDA in the dark for 45 min. Reactive oxygen species (ROS) were measured as an increase in fluorescence caused by the conversion of nonfluorescent dye to highly fluorescent 20,70-dichlorofluorescein, with an excitation wavelength of 488 nm and emission wavelength of 530 nm, in a fluorescence microplate reader (Victor X3, PerkinElmer).

### 2.8. Evaluation of Nile red accumulation

Epimastigotes (1 × 10^6^ cells/mL treated with 18.0 and 77.0 μM of **C4**) and trypomastigotes (1 × 10^7^ cells/mL treated with 45.0 and 230.0 μM of **C4**) of *T*. *cruzi* were incubated at 28°C and 37°C, respectively, for 24 h. After treatment, the parasites were washed twice in PBS, pH 7.4, and incubated with 10 μg/mL of Nile red in the dark for 30 min. Fluorescence was measured in a fluorescence microplate reader (Victor X3, PerkinElmer) and analyzed using an Olympus BX51 fluorescence microscope at an excitation wavelength of 485 nm and emission wavelength of 535 nm. The images were captured using an Olympus UC30 camera.

### 2.9. Exposure of phosphatidylserine

Phosphatidylserine exposure was detected using annexin-V FITC, a calcium-dependent phospholipid binding protein. Epimastigotes (5 × 10^6^ cells/mL treated with 18.0 and 77.0 μM of **C4**) and trypomastigotes (1 × 10^7^ cells/mL treated with 45.0 and 230.0 μM of **C4**) of *T*. *cruzi* were incubated at 28°C and 37°C, respectively, for 3 h. Afterward, the cells were washed and resuspended in 100 μL of binding buffer (140 mM NaCl, 5 mM CaCl_2_, and 10 mM HEPES-Na, pH 7.4), followed by the addition of 5 μL annexin-V FITC for 15 min at room temperature. Binding buffer (400 μL) and 0.2 μg/mL PI were then added. Data acquisition and analysis were performed using a FACSCalibur flow cytometer equipped with CellQuest software. A total of 10,000 events were acquired in the region that was previously established as the one that corresponded to the parasites. The following analyzes were performed: cells apoptotic (annexin V-positive—FL1, but PI-negative—FL2), late apoptotic cells (annexin V-positive—FL1, but PI-positive—FL2) and cells in necrosis (annexin V-negative—FL1, but PI-positive—FL2) [10].

### 2.10. Cell volume determination

Epimastigotes (5 × 10^6^ cells/mL treated with 18.0 and 77.0 μM of **C4**) and trypomastigotes (1 × 10^7^ cells/mL treated with 45.0 and 230.0 μM of **C4**) of *T*. *cruzi* were incubated at 28°C and 37°C, respectively, for 3 h. Afterward, the protozoa were collected by centrifugation, washed twice in PBS, and resuspended in PBS. Data acquisition and analysis were performed using a FACSCalibur flow cytometer equipped with CellQuest software. A total of 10,000 events were acquired in the region that was previously established as the one that corresponded to the parasites. Histograms and analyses were performed using CellQuest software. Forward light scatter (FSC-H) was considered to represent cell volume.

### 2.11. Evaluation of DNA fragmentation

DNA double-strand ruptures were analyzed *in situ* using a TUNEL kit. Epimastigotes (1 × 10^6^ cells/mL) were treated with 18.0 and 77.0 μM of **C4** for 24 h at 28°C, after the cells were subjected to the TUNEL assay according to the manufacturer’s instructions. Actinomycin D (10.0 μg/mL) was used as a positive control. Fluorescence was observed in an Olympus BX51 fluorescence microscope, and pictures were captured with an Olympus UC30 camera.

### 2.12. Cell membrane integrity

Epimastigotes (5 × 10^6^ cells/mL treated with 18.0 and 77.0 μM of **C4**) and trypomastigotes (1 × 10^7^ cells/mL treated with 45.0 and 230.0 μM of **C4**) of *T*. *cruzi* were incubated at 28°C and 37°C, respectively, for 3 h. Afterward, the parasites were washed with PBS and marked with 0.2 μg/mL PI for 10 min to verify possible alterations in cell membrane integrity. Digitonin (40 μM) was used as a positive control for cell membrane alterations. The material was kept on ice until analysis. Data acquisition and analysis were performed using a FACSCalibur flow cytometer equipped with CellQuest software. A total of 10,000 events were acquired in the region that was previously established as the one that corresponded to the parasites.

### 2.13. Statistical analysis

The data that are shown in the graphs are expressed as mean ± standard error (SE) of at least three independent experiments. The data were analyzed using two-way and one-way analysis of variance (ANOVA), with significant differences among means identified using the Bonferroni and Tukey *post hoc* tests. Values of *p*≤ 0.05 were considered statistically significant. The statistical analysis was performed using GraphPad software.

## Results

### 3.1. C4 induces mitochondrial depolarization

**Fig 2:**
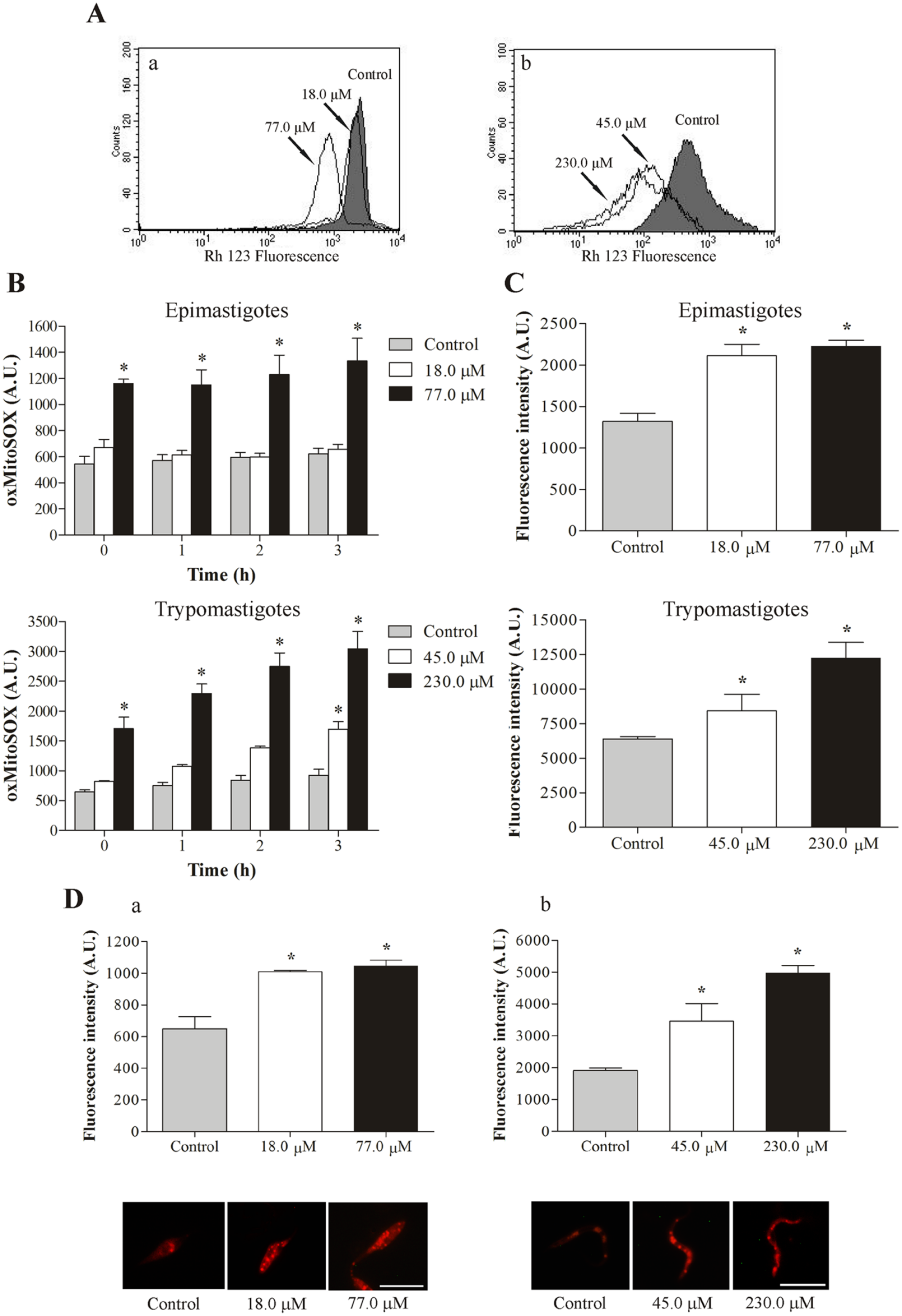
Evaluation of mitochondrial membrane potential, ROS production and lipid inclusions in *T*. *cruzi* treated with C4. **(A)** Mitochondrial depolarization in *T*. *cruzi* treated with **C4** for 3 h and stained with the fluorescence probe Rh 123. **(a)** Epimastigote forms. **(b)** Trypomastigote forms. Arrows correspond to the concentrations tested. The control group (untreated parasites) is also shown. **(B)** Mitochondrial O_2_^•−^ production in epimastigote and trypomastigote forms of *T*. *cruzi* treated with **C4** for up to 3 h. Mitochondrial O_2_^•−^ production was evaluated using the fluorescence probe MitoSOX. At the indicated times, oxidized MitoSOX (oxMitoSOX) was fluorimetrically measured in the parasites. **(C)** Total ROS production in epimastigote and trypomastigote forms of *T*. *cruzi* treaded with **C4** for 24 h and stained with the cell-permeable probe H_2_DCFDA. **(D)** Lipid inclusions in *T*. *cruzi* treated with **C4** for 24 h and stained with the fluorescence probe Nile red. **(a)** Epimastigote forms. **(b)** Trypomastigote forms. The images suggest a random distribution of lipid-storage bodies in the parasites. Scale bars = 10 μm. The data (**B**, **C** and **D**) are expressed as the mean fluorescence (in arbitrary units) ± SE. *p ≤ 0.05, significant difference compared with the control group (untreated parasites).

Based on previous studies that reported the effect of **C4** on *T*. *cruzi* mitochondria [6], we evaluated ΔΨm in **C4**-treated cells by flow cytometry. The histograms showed a noticeably pronounced loss of ΔΨm in both the epimastigote and trypomastigote forms of *T*. *cruzi* at the highest concentrations assayed after 3 h of treatment, with > 60.0% reductions of ΔΨm compared with the control group (Fig 2A). The positive control CCCP decreased ΔΨm by 50.7% and 76.3% in epimastigotes and trypomastigotes, respectively (data not shown).

### 3.2. C4 increases mitochondrial O_2_^•−^ production

Based on our ΔΨm results, we evaluated mitochondrial superoxide anion (O_2_^•−^) production. Fig 2B shows a significant increase in mitochondrial O_2_^•−^ production at the highest concentrations assayed for epimastigote and trypomastigote forms compared with the control group at all times tested. In epimastigotes that were treated with 77.0 μM of **C4**, we observed a 115.0% increase with 3 h of incubation. In trypomastigotes that were treated with 45.0 and 230.0 μM of **C4**, this increase was 84.0% and 230.0%, respectively, with 3 h of incubation. The positive control (AA) also increased mitochondrial O_2_^•−^ production (data not shown).

### 3.3. C4 increases total reactive oxygen species

In addition to mitochondrial O_2_^•−^ production, we evaluated the production of reactive oxygen species (ROS) in **C4**-treated parasites. Fig 2C shows that **C4** significantly increased total ROS production at both forms of *T*. *cruzi* after 24 h of treatment compared with the control group. In epimastigotes that were treated with 18.0 and 77.0 μM of **C4**, the increase in total ROS was 60.0% and 68.0%, respectively. In trypomastigotes that were treated with 45.0 and 230.0 μM of **C4**, the increase was 32.0% and 92.0%, respectively. The positive control (H_2_O_2_) also increased total ROS production (data not shown).

### 3.4. C4 induces lipid body formation

Epimastigotes and trypomastigotes of *T*. *cruzi* that were treated for 24 h with **C4** exhibited the presence of many lipid bodies marked with Nile red. Two assays showed this alteration: (*i*) fluorescence microscopy revealed the presence of lipid bodies, and (*ii*) the fluorimetric assay quantified this accumulation. These assays showed a concentration-dependent increase in the number of lipid bodies (Fig 2D), with an increase > 50% for epimastigotes and trypomastigotes at both concentrations tested.

### 3.5. C4 induces phosphatidylserine exposure

**Fig 3:**
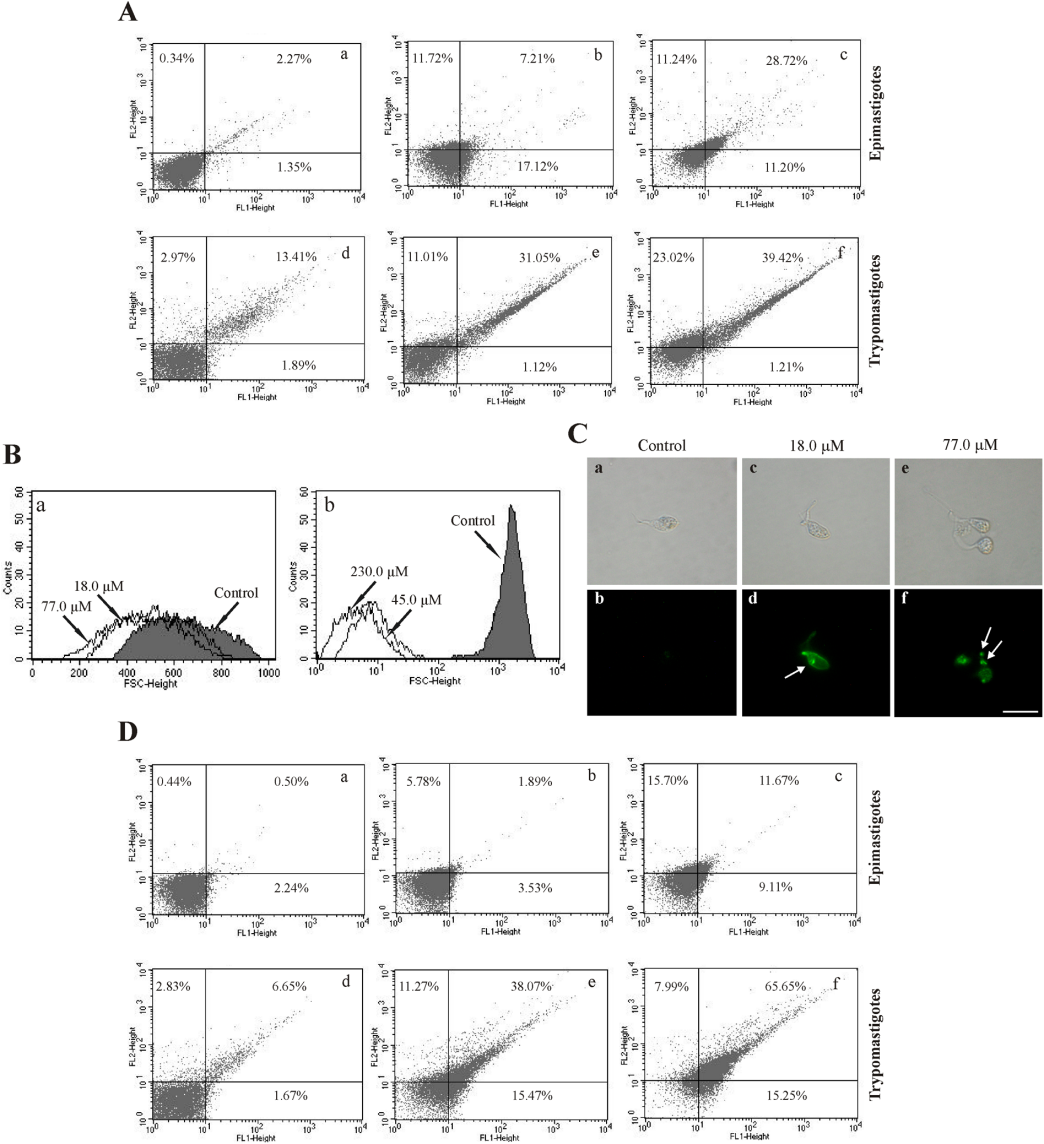
Evaluation of phosphatidylserine exposure, cell volume, DNA fragmentation and cell membrane integrity in *T*. *cruzi* treated with C4. **(A)** Phosphatidylserine exposure in epimastigote and trypomastigote forms of *T*. *cruzi* treated with **C4** for 3 h and stained with the fluorescence probes annexin V-FITC and PI. **(a)** Untreated epimastigote. **(b, c)** Epimastigotes treated with 18.0 and 77.0 μM of **C4**. **(d)** Untreated trypomastigotes. **(e, f)** Trypomastigotes treated with 45.0 and 230 μM of **C4**. **(B)** Reduction of cell volume of *T*. *cruzi* treated with **C4** for 3 h and analyzed by flow cytometry. **(a)** Epimastigote forms. **(b)** Trypomastigote forms. Forward light scatter (FSC-H) was considered to represent cell volume. Arrows correspond to the concentrations tested. The control group (untreated parasites) is also shown. **(C)** DNA fragmentation in epimastigote forms of *T*. *cruzi* treated with **C4** for 24 h and stained with TUNEL. The gray column indicates differential interference contrast, and the black column indicates fluorescence. **(a-b)** Untreated epimastigotes. **(c, d)** Epimastigotes treated with 18.0 μM. **(e, f)** Epimastigotes treated with 77.0 μM. Green fluorescence indicates DNA fragmentation. Scale bars = 10 μm. **(D)** Alteration of the cell membrane in epimastigote and trypomastigote forms of *T*. *cruzi* treated with **C4** for 3 h and stained with the fluorescence probe PI. (a) Untreated epimastigotes. (b, c) Epimastigotes treated with 18.0 and 77.0 μM of **C4**. (d) Untreated trypomastigotes. (e, f) Trypomastigotes treated with 45.0 and 230.0 μM of **C4**. The numbers show the percentage of PI-positive cells in the upper right and left quadrants.

Increases in ROS can lead to apoptosis-like cell death. Apoptosis is characterized by biochemical alterations, including phosphatidylserine exposure [11, 12]. We evaluated whether **C4** induces phosphatidylserine exposure. As shown in Fig 3A, epimastigote and trypomastigote forms that were treated with **C4** exhibited an increase in annexin-V fluorescence intensity after 3 h of treatment compared with the untreated parasites, indicating phosphatidylserine exposure. The histograms showed a > 30.0% increase in the intensity of annexin-V fluorescence at both concentrations tested for trypomastigote forms (Fig 3: e and f). For epimastigote forms, at the higher concentration, annexin-V fluorescence was observed in approximately 40.0% of the parasites (Fig 3: b and c).

### 3.6. C4 decreases cell volume

The present results indicate that **C4** induced phosphatidylserine exposure, and we explored the action of this compound on the apoptosis cell death pathway. We performed additional experiments to evaluate cell shrinkage, a hallmark of apoptotic death [12, 13]. As shown in Fig 3B, a decrease in cell volume was observed in trypomastigotes at both concentrations of **C4** tested after 3 h, with reductions of approximately 90.0% (Fig 3B: b). For epimastigotes, at the higher concentration, we observed a decrease in cell volume in approximately 20.0% of the parasites (Fig 3B: a).

### 3.7. C4 induces DNA fragmentation

Continuing the same line of reasoning, we then evaluated possible cell death by apoptosis, reflected by DNA fragmentation, using the TUNEL assay. Fig 3C illustrates the analysis of DNA fragmentation, which was performed following treatment of the parasites with different concentrations of **C4** for 24 h. Epimastigotes that were treated with 18.0 and 77.0 μM of **C4** (Fig 3C: d and f, respectively) exhibited bright fluorescence, indicating DNA double-strand ruptures compared with untreated parasites. Bright fluorescence was also observed with the positive control (actinomycin D; data not shown).

### 3.8. C4 induces alterations in cell membrane integrity

Previous work also demonstrated the effect of **C4** on the cell membrane [6]. We further evaluated the effect of **C4** on membrane integrity in epimastigote and trypomastigote forms of *T*. *cruzi*. **C4** affected membrane integrity in both forms of *T*. *cruzi* after 3 h of treatment compared with untreated parasites. The histograms showed an increase in the intensity of PI fluorescence at both concentrations tested (49.34% and 73.64% PI-positive parasites in Fig 3D: e and f), mainly for trypomastigotes, indicating alterations in cell membrane integrity. In epimastigotes at the higher **C4** concentrations, approximately 27% of the parasites were PI-positive (Fig 3D: c). The positive control (digitonin) increased fluorescence by 41.02% and 93.82% in epimastigotes and trypomastigotes, respectively (data not shown).

## Discussion

β-carbolines have presented numerous biological properties, such as antimicrobial [14], antitumoral [15], antiviral [16] and antiparasitic [6–8] effects. In our recent studies, we demonstrated the *in vitro* and *in vivo* activity of **C4** against *T*. *cruzi* [6, 7]. Additionally, **C4** induced low cytotoxicity, with a selective index higher to the parasites than for mammalian cells [6]. In the present study, we focused on elucidating the mechanism of action of **C4** in the cell death of epimastigotes and trypomastigotes of *T*. *cruzi*.

Our previous study reported ultrastructural alterations, especially in the mitochondria, in parasites that were treated with **C4** [6]. The present results confirmed that mitochondria are a target of **C4**, reflected by the depolarization of ΔΨm and increase in the production of mitochondrial ROS and formation of lipid droplets in parasites treated with **C4**. Changes in ΔΨm are associated with opening of the permeability transition pore (PTP) in the mitochondrial membrane [17, 18]. Thus, **C4** might induce the opening of PTP in the mitochondrial membrane, leading to activation of the apoptotic pathway [19]. This programmed cell death is commonly characterized by different morphological characteristics, such as exposure of phosphatidylserine residues on the external leaflet of the cell membrane, decrease of cell volume and DNA fragmentation [20]. In addition, analysis of red Nile showed an increase of lipid bodies in the cytoplasm, which may indicate that the **C4** changes the content of phospholipids and sterols of *T*. *cruzi*, and is strongly related to mitochondrial dysfunction [21]. Previous studies with an alkyl phosphocholine-dinitroaniline hybrid molecule [22] and antifungal azoles [23] showed similar results.

Besides acting in the mitochondrial membrane, the **C4** also acts on the plasmatic membrane of the parasites. This can be seen in the results obtained starting the labeling of parasites with PI, and also by increase in population of PI-positive parasites/annexin-V negative in the upper left quadrant in relation to the control. Morphological alterations in the plasma membrane are features of cell death by necrosis [20]. However, the increase of cellular ROS production might induce different mechanisms of cell death including both apoptosis and necrosis [24] which can occur in the same population of cells. Similar results have been described for other compounds (e.g., eupomatenoid-5 in *T*. *cruzi* parasites [25] and 4-nitrobenzaldehyde thiosemicarbazone, derived from S-limonene in *L*. *amazonensis* [26].

Our results demonstrated that **C4** can induce several changes in the parasites that lead to cell death either by apoptosis or necrosis [12, 27–29]. Our results suggest that mitochondria are one of the target organelles that may be involved in the increase in ROS production through the electron transport chain, which affects cellular structures and induces parasite death. Altogether, the present results suggest that new chemotherapeutic agents can be developed for the treatment of Chagas’ disease.
